# Comprehensive Transcriptome-Wide Profiling of 5-Methylcytosine Modifications in Long Non-Coding RNAs in a Rat Model of Traumatic Brain Injury

**DOI:** 10.3390/cimb46120871

**Published:** 2024-12-23

**Authors:** Zhijun Xiang, Yixing Luo, Jiangtao Yu, Haoli Ma, Yan Zhao

**Affiliations:** 1 Emergency Center, Zhongnan Hospital of Wuhan University, 169 Donghu Road, Wuhan 430071, China; xxxzzj@whu.edu.cn (Z.X.); louyixing@whu.edu.cn (Y.L.); yjt@whu.edu.cn (J.Y.); 2 Department of Biological Repositories, Zhongnan Hospital of Wuhan University, Wuhan 430071, China; 3 Hubei Clinical Research Center for Emergency and Resuscitation, Zhongnan Hospital of Wuhan University, 169 Donghu Road, Wuhan 430071, China

**Keywords:** TBI, 5-methylcytosine, lncRNA, MeRIP-seq, RNA-seq

## Abstract

Traumatic brain injury (TBI) poses a major global health challenge, leading to serious repercussions for those affected and imposing considerable financial strains on families and healthcare systems. RNA methylation, especially 5-methylcytosine (m^5^C), plays a crucial role as an epigenetic modification in regulating RNA at the level of post-transcriptional regulation. However, the impact of TBI on the m^5^C methylation profile of long non-coding RNAs (lncRNAs) remains unexplored. In the present study, we conducted a thorough transcriptome-wide examination of m^5^C methylation in lncRNAs in a rat TBI model utilizing MeRIP-Seq. Our results revealed significant differences in the amount and distribution of m^5^C methylation in lncRNAs between TBI and control groups, indicating profound changes in m^5^C methylation following TBI. Bioinformatic analyses linked these specifically methylated transcripts to pathways involved in immune response, neural repair, and lipid metabolism, providing insight into possible mechanisms underlying TBI pathology. These findings offer novel perspectives on the post-transcriptional modifications in lncRNA m^5^C methylation following TBI, which may contribute to understanding the disease mechanisms and developing targeted therapeutic strategies.

## 1. Introduction

Traumatic brain injury (TBI) is a common disorder caused by trauma. The World Health Organization (WHO) predicts that TBI will overtake numerous other diseases as one of the most significant contributors to mortality and morbidity in the near future [1]. Each year, over 10 million individuals worldwide are hospitalized due to TBI [2]. This condition, also known as intracranial injury, primarily results from falls, traffic accidents, violence, and sports injuries, leading to both immediate and delayed motor and cognitive impairments, and even lifelong disabilities [3]. Individuals with TBI may encounter a broad spectrum of injuries that can have both short- and long-term effects. Over 50% of severe TBI survivors are left with significant disabilities within a year of their injury, with some enduring severe neurological impairments, including memory or cognitive impairments, as well as motor, sensory, or emotional dysfunctions [4]. These issues are common among TBI patients and can persist for decades after the injury [5].

Numerous studies have identified epigenetic changes as crucial components in the pathophysiological responses triggered by traumatic brain injury (TBI) [6]. Epigenetics encompasses the modulation of gene expression without modifying the DNA sequence itself [7]. Various chemical modifications naturally take place on cytosolic RNAs, impacting their formation, stability, and roles [8]. To date, the scientific community has identified and characterized over 150 distinct types of RNA modifications [9]. Among the various epigenetic mechanisms, RNA methylation represents a particularly noteworthy phenomenon, playing a crucial role in the post-transcriptional regulation in RNAs. It has emerged as a major area of research interest, encompassing several types, including m^6^A, m^1^A, m^5^C, m^7^G, among others. Specifically, RNA m^5^C applies to the methylation of cytosine at the fifth carbon position in RNA [10,11,12]. 5-Methylcytosine (m^5^C), which is present in RNA, is a common modifier in human RNAs [13]. Up to now, a total of 95,391 m^5^C peaks have been reported within the human metagenome [14]. In recent years, several related studies have demonstrated the pervasive presence of RNA m^5^C methylation in cells and its significant function in a wide variety of physiological and pathological procedures [15,16,17]. The m^5^C modification enhances RNA stability and reduces susceptibility to degradation [18]. It also promotes translation by facilitating ribosome binding. Research has demonstrated that m^5^C modifications in tRNA and mRNA help stabilize RNA secondary structures, leading to increased protein synthesis efficiency [19]. Furthermore, studies indicate that m^5^C modifications support cellular adaptation to oxidative stress and other challenging environmental conditions [20].

LncRNAs are a type of RNA molecules that are more than 200 nucleotides in length and originate from non-coding regions of the genome [21]. They are believed to be similar in quantity to protein-coding genes, though typically containing fewer exons, are shorter in length, and are less evolutionarily conserved [22,23]. Approximately 14,000 lncRNA genes in humans annotated based on the latest version of GenCode (version 19) [24]. LncRNAs have attracted considerable interest for their roles as crucial regulators of gene expression in diverse biological processes [25].

The functions of lncRNA m^5^C methylation in TBI has not been clearly understood until now. To investigate the epigenetic alterations and changes in m^5^C methylation in lncRNAs in TBI, we conducted a comprehensive whole-transcriptome analysis of m^5^C methylation profiles in TBI-affected rats using MeRIP-Seq. Furthermore, we conducted a GO analysis on significantly altered lncRNAs to predict their potential functions, providing a reference for future related studies.

## 2. Materials and Methods

### 2.1. Construction of Rat Traumatic Brain Injury Model

All experimental procedures complied with the National Institutes of Health Guide for the Care and Use of Laboratory Animals. The animal experiment was approved by the Animal Experimentation Center and Ethics Committee of Zhongnan Hospital of Wuhan University (IACUC: ZN2021119). Sprague Dawley rats with a mean weight of 250 to 300 g were conditioned for a minimum of 7 days prior to the initiation of the animal model, housed in an animal facility under controlled temperature (22–25 °C), humidity (50% relative humidity), and a 12 h light/dark cycle. The rats were made available to consume food and water at will. Rats were randomly divided into two groups of three rats in each group. Two additional rats underwent hematoxylin and eosin (HE) staining to evaluate the effectiveness of the TBI modeling procedure. A moderate TBI (mTBI) model was created with a weight-drop apparatus, as described in our previous studies [26,27]. Simply, rats were anesthetized using 1% pentobarbital by intraperitoneal injection at a dose of 30 mg/kg. After anesthesia was completed, the surgical area of the rats was shaved and then sterilized three times using 0.5% iodine. Under sterile conditions, the skull was exposed, and a borehole (5 mm in diameter) was drilled on the right side to expose the dura mater. A 50 g weight was then dropped from 25 cm above onto the uncovered brain tissue to induce TBI. The rats both in the Sham and the TBI group used the same procedure with the exception of the step of impacting the brain tissue with the 50 g weight.

After surgery, the rats were kept separately in individual cages until they recovered from anesthesia. Furthermore, the rats were permitted to consume food and water at their discretion. After 24 h, the rats were euthanized via an intraperitoneal injection of sodium pentobarbital (100 mg/kg). Following euthanasia, the brain tissue was carefully dissected under aseptic conditions, immediately placed in liquid nitrogen. Subsequently, the samples were transferred to an ultra-low temperature freezer set at −80 °C.

### 2.2. Hematoxylin and Eosin Staining

The rat brain tissues were preserved in 4% paraformaldehyde and encased in paraffin wax. Thin serial longitudinal sections (5 μm) were sliced from the paraffin blocks and analyzed histopathologically with HE staining under a light microscope.

### 2.3. MeRIP-Seq

Using the GenSeq^®^ m^5^C-IP Kit (GenSeq Inc., Dublin, Ireland), the manufacturer’s instructions were strictly followed for all immunoprecipitation reactions. The process involved randomly fragmenting RNA into approximately 200 nucleotide pieces to bind the m^5^C antibody to Protein A/G beads. Antibodies were incubated for 1 h at ambient temperature. Subsequently, the RNA fragments were incubated with the bead-bound antibodies for 4 h at 4 °C to facilitate RNA binding to the antibody. After several washes, the RNA-antibody complexes were eluted and purified to isolate the captured RNA. RNA libraries for immunoprecipitation (IP) and input samples were then prepared using the GenSeq^®^ Low Input Whole RNA Library Prep Kit (GenSeq Inc.) according to the manufacturer’s instructions. The quality of the libraries was assessed using an Agilent 2100 Bioanalyzer, and then the sequencing was conducted on a NovaSeq platform (Illumina, San Diego, CA, USA).

### 2.4. RNA Library Preparation and Sequencing (RNA-Seq)

The process began by removing ribosomal RNA (rRNA) from the samples using the GenSeq^®^ rRNA Removal Kit (GenSeq Inc.). The rRNA-depleted samples were then prepared for library construction with the GenSeq^®^ Low Input RNA Library Prep Kit. According to the manufacturer’s instructions, the libraries’ quality and quantity were assessed using the BioAnalyzer 2100 system (Agilent Technologies Inc., Santa Clara, CA, USA). Finally, sequencing was carried out on an Illumina NovaSeq platform with 150 base pair paired-end reads.

## 3. Data Analysis

To generate the raw reads, image annotation, base reading, and quality control were performed. The quality control assessment was by means of Q30 metrics. Following the removal of 3′ adapters and low-quality reads using Cutadapt (v1.9.3) [28], the processed reads were prepared for downstream analysis.

The initial step in RNA-seq data evaluation was the mapping of the high-quality sequencing reads to the relevant reference genome using the Hisat2 (version 2.0.4) [29]. Subsequently, raw transcript-level counts were obtained as lncRNA expression profiles with HTSeq (v0.9.1) [30]. LncRNAs with differential expression were screened using edgeR (v3.16.5) [31], which normalized the data and calculated the fold change and *p*-value between the two sets of samples. Following this step, predicted lncRNA target genes were identified, and Gene Ontology (GO) analyses were conducted for these target genes.

The next step was the analysis of MeRIP-Seq. Initially, clean reads from the input libraries were mapped to the genome (UCSC rn6) using STAR software (v2.7.9a) [32]. Subsequently, clean reads from all of the libraries were mapped to the target Genome using the Hisat2 software (v2.0.4) [29]. The identification of methylation peaks was with MACS software (v2.2.7.1) [33], and peaks that are aberrantly methylated were determined using diffReps [34]. In both software, these peaks were identified as overlapping lncRNA exons and were assigned to and selected by custom scripts. Gene Ontology (GO) analyses were then performed for protein-encoding genes with differential methylation and for genes associated with aberrantly methylated lncRNAs.

## 4. Results

**Figure 1:**
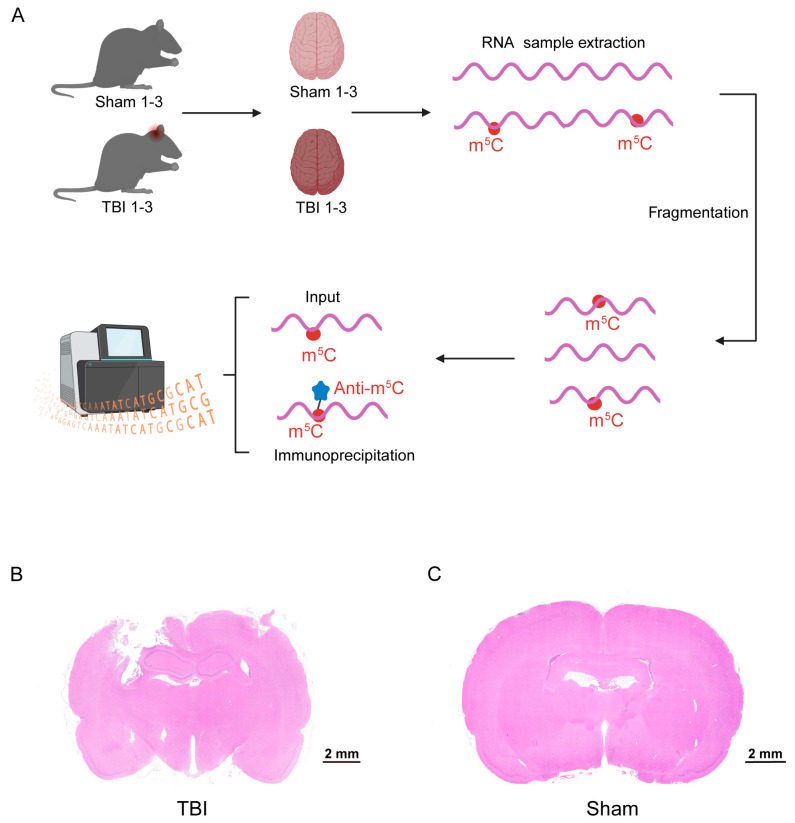
**Experimental procedure:** (**A**). The general process of the experiment; (**B**). HE staining of coronal brain slices from the rat TBI model; and (**C**). HE staining of coronal sections of brain tissue from the sham group.

Given the absence of relevant studies, it remains unclear whether the m^5^C methylation profile of long non-coding RNAs (lncRNAs) is altered following traumatic brain injury (TBI). To address this uncertainty, we conducted a comprehensive transcriptome analysis of m^5^C methylation in lncRNAs using MeRIP-Seq in the rat model of TBI. A flowchart summarizing the experimental procedure is shown in (Figure 1A). Twenty-four hours after establishing the TBI rat model, the rats were euthanized, and the intact brain tissues were collected under aseptic conditions for use in subsequent experiments. Notably, significant brain parenchymal damage was observed in the rats following TBI model construction (Figure 1B), whereas the brain parenchyma in the sham-operated group remained intact (Figure 1C).

### 4.1. General Characteristics of m^5^C Methylation in TBI Rat Model

**Figure 2:**
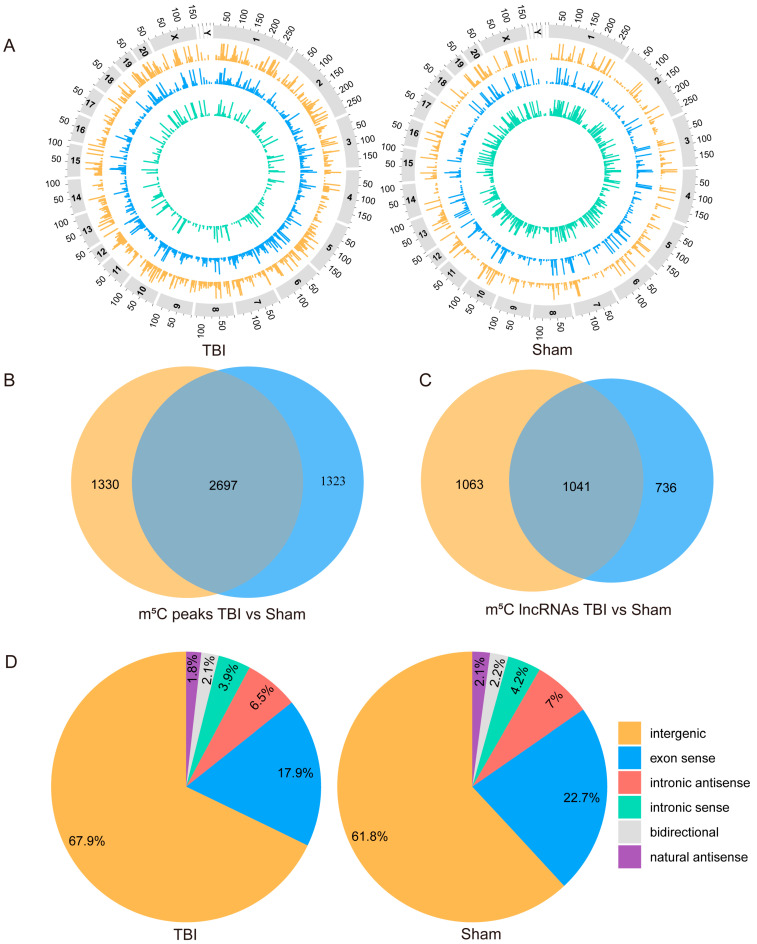
**Overview of lncRNA m^5^C methylation in both sham and TBI groups:** (**A**). Visualization of m^5^C peaks at the chromosomal level in TBI and sham groups. (**B**). Venn diagram showing the number of m^5^C methylation peaks detected in lncRNAs in TBI and sham groups. (**C**). Venn diagram showing the number of lncRNAs with m^5^C peaks in TBI and sham groups. (**D**). Pie charts depicting the distribution of methylated lncRNA sources in TBI and sham groups.

We conducted a genome-wide analysis of the distribution of m^5^C methylation peaks in TBI and control mice. It was evident that m^5^C methylation peaks in rats are widely distributed across all chromosomes in both the TBI and control groups, with the lowest density observed on the Y chromosome. (Figure 2A). The sequencing results revealed a total of 4027 m^5^C methylation peaks in the lncRNAs of TBI rats, while 4020 peaks were detected in the control rats. Of these, 1330 peaks were unique to the TBI, and 1323 peaks were specific to the sham (Figure 2B). Upon further analysis, we found that m^5^C methylation modification was detected on a total of 2104 lncRNAs in the TBI group, while it was present on a total of 1777 lncRNAs in the sham group. There were 1041 lncRNAs with m^5^C methylation modifications in both TBI and sham groups (Figure 2C). We next analyzed the specific m^5^C methylation peaks on lncRNAs and found that these peaks were distributed across all substructures of lncRNAs. A majority of the methylation peaks were located in intergenic regions, with 67.9% in the TBI group and 61.8% in the sham group (Figure 2D). Furthermore, we observed an increase in the percentage of methylated peaks in the intergenic regions of lncRNAs following TBI, whereas the exon sense regions exhibited a decreased percentage of methylated peaks compared to the sham group (*p* < 0.05) (Supplementary S1).

### 4.2. Peak Alterations After Traumatic Brain Injury

**Figure 3:**
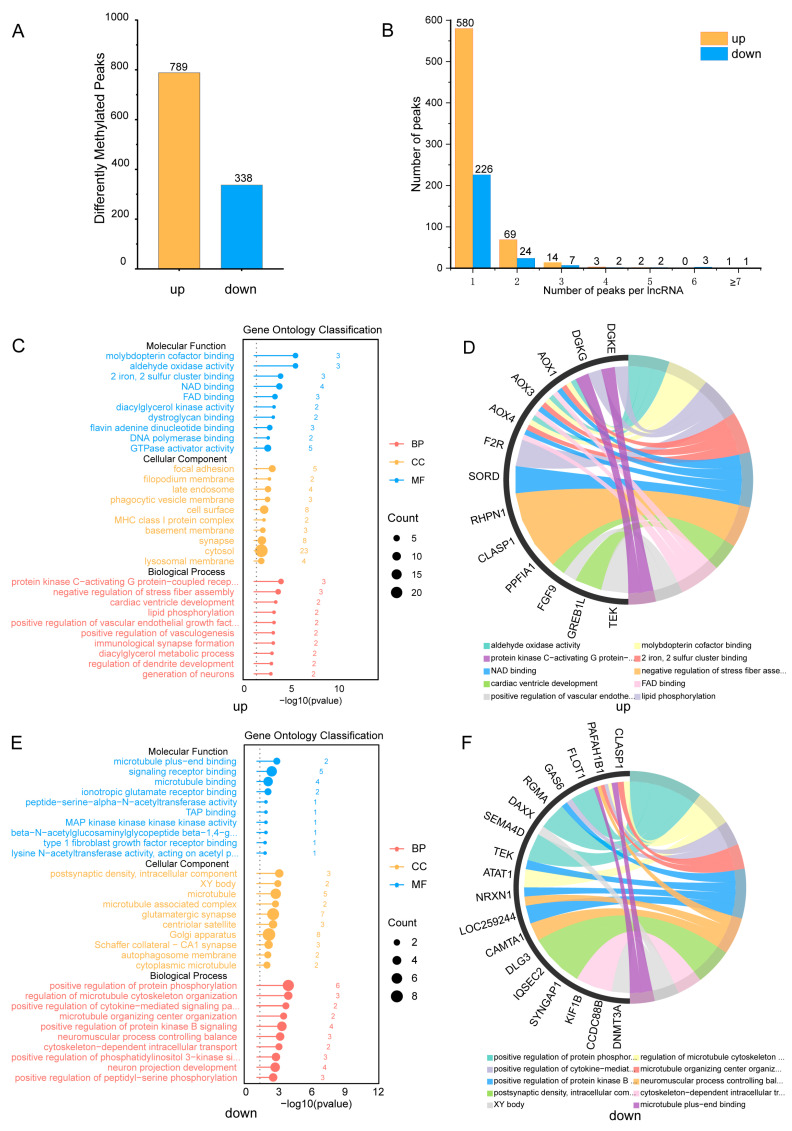
**Characteristics of post-TBI lncRNA m^5^C methylation and GO analysis:** (**A**). Number of significantly up-regulated and down-regulated m^5^C peaks in TBI rats (*p* < 0.05, FC > 2). (**B**). Number of m^5^C peaks on each lncRNA in TBI and sham rats (*p* < 0.05, fold change > 2). (**C**). Significantly enriched GO categories for hyper-methylated lncRNAs. *Incomplete GO term displayed: BP: Protein kinase C-activating G protein-coupled receptor signaling pathway; Positive regulation of vascular endothelial growth factor receptor signaling pathway.* (**D**). String diagrams showing the connections between different GO categories of hyper-methylated lncRNAs. (**E**). Significantly enriched GO categories for hypo-methylated lncRNAs. *Incomplete GO term displayed: MF: Beta-N-acetylglucosaminylglycopeptide beta-1,4-galactosyltransferase activity; Lysine N-acetyltransferase activity, acting on acetyl phosphate as donor. BP: positive regulation of cytokine-mediated signaling pathway*; positive regulation of phosphatidylinositol 3-kinase signaling pathway. (**F**). String diagrams showing the connections between different GO categories of hypo-methylated lncRNAs.

We further analyzed the differential m^5^C peaks to investigate alterations in m^5^C methylation after TBI. A total of 1127 m^5^C methylation significantly altered peaks were identified in the TBI group compared to the sham rats, including 789 up-regulated and 338 down-regulated m^5^C peaks (*p* < 0.05, FC > 2) (Figure 3A). Further analysis of these methylation peaks revealed that m^5^C methylation peaks and lncRNAs are not always in a one-to-one relationship; multiple methylation peaks can occur on the same lncRNA. However, in most cases, there is only one m^5^C methylation peak on a single lncRNA. Of the 789 up-regulated methylation peaks, 580 were located on individual lncRNAs, indicating that 580 lncRNAs had only one m^5^C peak. In addition, 69 lncRNAs had two methylation peaks, with a maximum of nine peaks found on a single lncRNA. For the down-regulated peaks, 226 lncRNAs had only one m^5^C peak, while 24 lncRNAs had two methylation peaks, with up to 15 m^5^C methylation peaks occurring on a single lncRNA (Figure 3B). To elucidate the functionality of these distinctively methylated genes, a GO analysis (http://www.geneontology.org (accessed on 8 November 2024)) was performed [35,36], which was on the basis of differentially methylated genes that were analyzed to determine their associated biological processes (BPs), cellular components (CCs), and molecular functions (MFs). A *p*-value less than 0.05 was considered the significant GO terms. For lncRNAs with hyper-methylated peaks, we identified a total of 869 GO terms (Supplementary S2) and presented the top ten terms for each category (Figure 3C). For the BP, they were enriched at the protein kinase C-activating (PKC) G protein-coupled receptor (GPCR) signaling pathway, the negative regulation of stress fiber assembly, the positive regulation of the vascular endothelial growth factor receptor (VEGFR) signaling pathway, and neuron generation, which are related to the regulation of TBI mechanisms. For the MF, genes with up-methylated m^5^C peaks were notably enriched in activities such as the GTPase activator activity, the aldehyde oxidase activity, molybdopterin cofactor binding, and 2 iron, 2 sulfur cluster binding. For the CC, genes with up-methylated m^5^C peaks were mainly enriched in the focal adhesion, filopodium membrane, late endosome, phagocytic vesicle membrane, etc. For lncRNAs with hyper-methylated peaks, we identified a total of 789 GO terms (Supplementary S3) and presented the top ten terms for each category (Figure 3E). The BP was enriched in the positive regulation of protein phosphorylation, the positive regulation of the cytokine-mediated (JAK-STAT) signaling pathway, the positive regulation of phosphatidylinositol 3-kinase (PI3K) signaling pathway, and so on. The CC was enriched in the postsynaptic density, intracellular component, XY body and microtubule, etc. The MF was enriched in the microtubule plus-end binding, the signaling receptor binding, the microtubule binding, etc. Subsequently, we demonstrate the interrelationship between distinct GO categories and genes (Figure 3D,F). Then, we listed the top 10 lncRNAs with the greatest differences in up-regulated and down-regulated methylation enrichment levels (Table 1).

### 4.3. Modification of lncRNA Expression Profile After TBI

**Figure 4:**
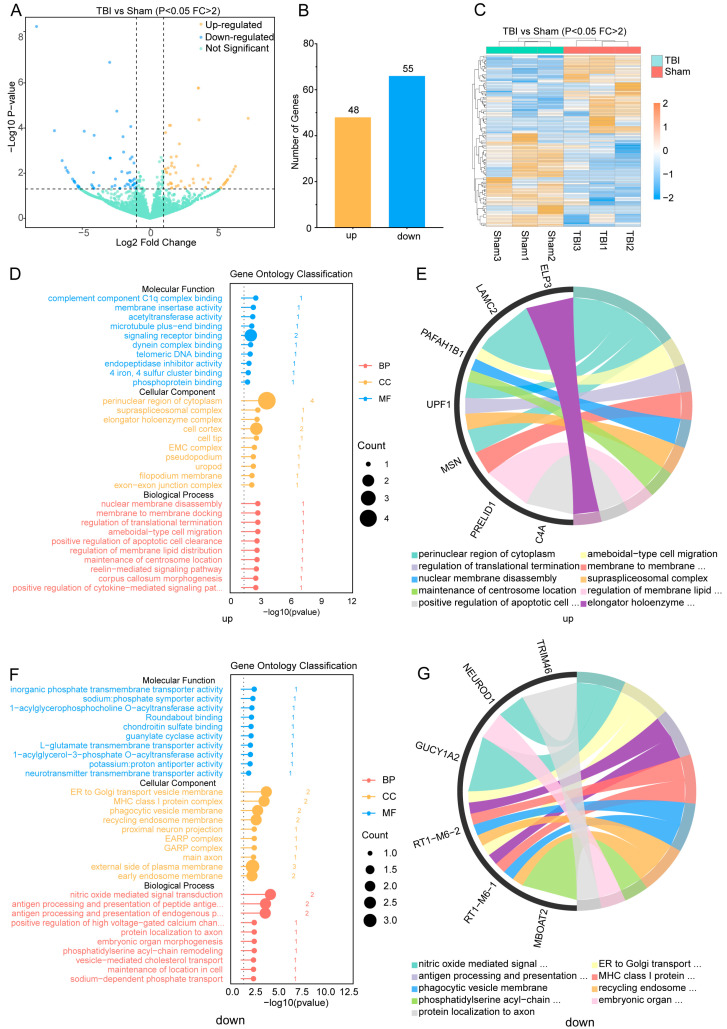
**Changes in lncRNA expression and GO analysis after TBI:** (**A**). Volcano plot displaying the lncRNAs that were significantly up-regulated and down-regulated after TBI (fold change > 2, *p*-value < 0.05). (**B**). Number of up-regulated and down-regulated lncRNAs (fold change > 2, *p*-value < 0.05). (**C**). Cluster analysis of differentially expressed lncRNAs. (**D**). Significantly enriched GO categories for up-regulated lncRNAs. *Incomplete GO term displayed: BP: positive regulation of cytokine-mediated signaling pathway.* (**E**). String diagrams showing the connections between different GO categories of the up-regulated lncRNAs. (**F**). Significantly enriched GO categories for down-regulated lncRNAs. *Incomplete GO terms displayed: BP: antigen processing and presentation of peptide antigen via MHC class I; antigen processing and presentation of endogenous peptide antigen via MHC class Ib; positive regulation of high voltage-gated calcium channel activity.* (**G**). String diagrams showing the connections between different GO categories of the down-regulated lncRNAs.

Following TBI, substantial changes were observed not only in the methylation but also in the expression profiles of lncRNAs. Our sequencing results confirmed these findings, identifying a total of 1487 up-regulated and 1600 down-regulated lncRNAs (Figure 4A). Among these, 103 genes showed significant differential expression, with 48 up-regulated and 55 down-regulated genes (Fold change > 2, *p* < 0.05) (Figure 4B). The heatmap displays the relative expression levels of each sample in the TBI and sham groups, where samples within the same group exhibited similar patterns, while distinct patterns were observed between the two groups (Figure 4C). The GO enrichment analysis uncovered possible roles for lncRNAs that were expressed differently. For genes with hyper-methylation, the BPs were enriched in nuclear membrane disassembly, the positive regulation of the JAK-STAT signaling pathway, and the reelin-mediated (RM) signaling pathway. The CCs were enriched in the perinuclear region of the cytoplasm, the supraspliceosomal complex, and the elongator holoenzyme complex. The MFs were enriched in signaling receptor binding, acetyltransferase activity, and endopeptidase inhibitor activity (Figure 4D). We further demonstrated the interrelationships between distinct GO categories and genes (Figure 4E). Detailed GO terms are provided in the supplementary file (Supplementary S4). For genes with hypo-methylation, the BPs were enriched in nitric oxide-mediated signal transduction, phosphatidylserine acyl-chain remodeling, and neurotransmitter loading into synaptic vesicles. The CCs were enriched in proximal neuron projection, the axon initial segment, and presynaptic active zone. The MFs were enriched in guanylate cyclase activity, L-glutamate transmembrane transporter activity, and neurotransmitter transmembrane transporter activity (Figure 4F). We also demonstrated the interrelationships between different GO categories and genes (Figure 4G). Detailed GO terms are provided in the supplementary file (Supplementary S5). Subsequently, we listed the 20 lncRNAs with the most significantly different expression levels, showing the top up-regulated and down-regulated lncRNAs (Table 2).

### 4.4. Conjoint Analysis of m^5^C Methylation and lncRNA Expression

**Figure 5:**
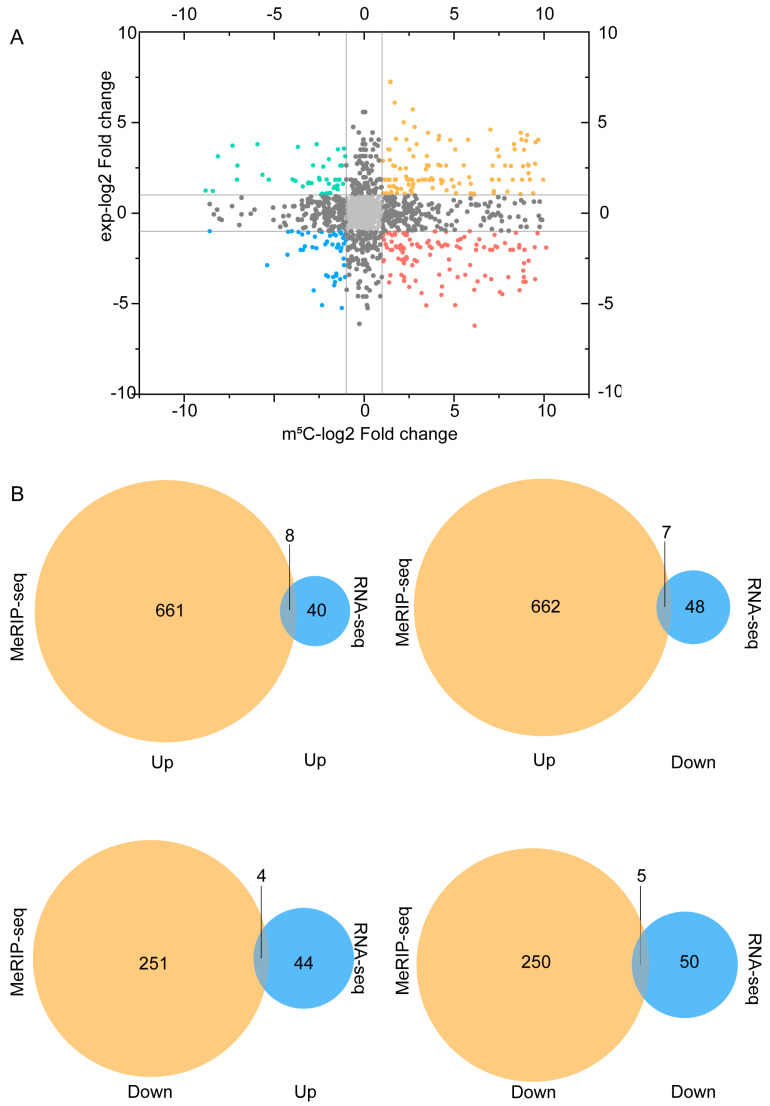
**Combined analysis of m^5^C methylation and lncRNA expression after TBI:** (**A**). The nine-quadrant plot illustrates the correlation between lncRNA m^5^C methylation and the corresponding lncRNA expression. Top-right quadrant: both methylation and gene expression are up-regulated (m^5^C-fold change > 2 and Exp-fold change > 2). Top-left quadrant: methylation is down-regulated while gene expression is up-regulated (m^5^C-fold change < −2 and Exp-fold change > 2). Bottom-left quadrant: both methylation and gene expression are down-regulated (m^5^C-fold change < −2 and Exp-fold change < −2). Bottom-right quadrant: methylation is down-regulated while gene expression is up-regulated (m^5^C-fold change > 2 and Exp-fold change < −2). (**B**). Venn diagrams showing the overlap between the number of differentially methylated genes and the number of differentially expressed lncRNAs.

To further investigate how m^5^C methylation relates to gene expression, this study involved a simultaneous examination of lncRNAs with alterations in methylation and expression. A nine-quadrant plot demonstrates the fold change of m^5^C methylated and expressed lncRNAs (Figure 5A). Through additional analysis, Venn diagrams were created to illustrate the correlation between differentially methylated genes and lncRNAs with differential expression (Figure 5B). We identified a set of eight hyper-methylated genes that were up-regulated (Table 3). Seven hyper-methylated genes were down-regulated (Table 4). Four hypo-methylated genes were up-regulated (Table 5), and five hypo-methylated genes were down-regulated (Table 6). (Fold change > 2, *p* < 0.05).

## 5. Discussion

TBI is frequently observed in sports-related injuries, military settings, and home environments. TBI can cause substantial damage to neuronal function and cognitive abilities, with these adverse effects potentially lasting for years or even a lifetime after the initial incident [37,38]. Over 1 million new TBI cases are reported every year in China [39]. A major challenge in developing diagnostic, therapeutic, and prognostic strategies for TBI is the complexity of its pathology. Epigenetics plays a significant role in traumatic brain injuries, as demonstrated by an increasing amount of research [40,41,42]. Gene expression studies are essential for uncovering the molecular mechanisms that contribute to TBI pathophysiology. For example, an RNA-Seq analysis of hippocampal and leukocyte samples from rats with TBI identified 240 and 1052 genes with differential expression, and 111 and 739 transcripts that are differentially expressed, respectively [40]. LncRNAs play a variety of roles in the regulation of gene expression at a number of different levels, such as epigenetic, transcriptional, post-transcriptional, and chromatin remodeling, by interacting with the 3′ untranslated region (UTR) of mRNAs [43]. The regulation of gene expression by lncRNAs is critical, and they are associated with a range of CNS disorders [44]. The expression of lncRNAs also changes significantly [41,45].

Numerous studies have demonstrated that RNA methylation is significantly altered in diseases associated with central nervous system (CNS) injury. For instance, changes in mRNA m^6^A methylation have been observed following traumatic spinal cord injury in rats [46]. The m^5^C modification is abundant in non-coding RNAs, particularly in long non-coding RNAs, and is implicated in the regulation of gene expression by modulating the RNA secondary structure. This modification may thus play a pivotal role in cellular differentiation, development, and the pathogenesis of various diseases. In non-coding RNAs, m^5^C modifications are postulated to operate through mechanisms such as gene silencing regulation, chromatin architecture remodeling, and the enhancement of protein-RNA interactions [16,18]. The role of m^5^C modification in cancer has become an important research area; abnormal m^5^C modifications have been found in various cancers, particularly in mRNA and non-coding RNA [47]. However, there is currently no compelling evidence to suggest that m^5^C impacts the expression levels of lncRNAs.

To date, there have been no studies on the altered m^5^C methylation profile lncRNA after TBI; our study addresses this gap. Some studies suggest that lncRNAs have a significant role in mediating diseases of the central nervous system. For example, Ginkgo biloba extract ameliorates inflammatory responses and reduces neuronal damage in the hippocampus of status epilepticus mice by down-regulating lncRNA-COX2/NF-κB signaling, which improves memory function in mice [43]. Additionally, LINC00938 mitigates neonatal brain injury caused by hypoxic–ischemic encephalopathy by regulating oxidative stress and inhibiting the JNK/p38 MAPK pathway [48]. The involvement of lncRNAs in the pathological process of TBI through different pathways has been confirmed by several studies. An RNA-Seq study on mice after TBI showed 667 lncRNAs were up-regulated, while 156 lncRNAs were down-regulated [49]. Furthermore, the number of altered lncRNAs in human TBI expression profiles were associated with the severity of TBI. Specifically, a greater number of lncRNA alterations were observed in cases of more severe injury [45].

We determined the m^5^C methylation profile of lncRNAs in TBI rats using MeRIP-seq, which provides evidence for further study of the association between lncRNA m^5^C methylation and TBI. Our study identified 1127 m^5^C methylation peaks that were significantly altered, among which 789 peaks were up-regulated and 338 were down-regulated. The distribution and the number of these peaks were significantly different between the TBI and the normal rat samples, which provides direct evidence for a clear link between m^5^C methylation alterations and TBI. Compared to normal rats, our RNA-seq results showed that a total of 48 lncRNAs were up-regulated and 55 lncRNAs were down-regulated in TBI rats (Fold change > 2, *p* < 0.05). Results may vary slightly depending on the severity of the TBI and the species used for the experiment. We then conducted a cross-linking analysis of lncRNA methylation and expression profiles. We found significant changes in m^5^C methylation and lncRNA expression after TBI in rats, further supporting a potential link between m^5^C methylation and TBI.

Subsequently, we conducted a GO analysis to investigate the functional roles of these lncRNAs with distinct methylation patterns. The results showed that differentially methylated lncRNAs were primarily associated with pathways such as the GPCR signaling pathway, VEGFR signaling pathway, CMSP signaling pathway, and PI3K signaling pathway, as well as processes related to lipid metabolism, transport, phosphorylation, immune processes, and neural repair. Comparing our GO analysis results with the existing literature, we found many similarities. For instance, a study on a GO analysis of differentially expressed lncRNAs in mice post-TBI primarily involved immune processes, stress responses, the activation of biological and cellular processes, and receptor or cytokine binding and activation [49], which are highly consistent with our findings. These findings are also related to physiological processes within the nervous system, such as immune synapse formation, neuron generation, neuron projection development, and neurotransmitter transmembrane transport. Angiogenesis describes the process of generating new blood vessels from preexisting established vascular networks [50]. The process of angiogenesis leads to the development of new blood vessels, thereby providing the brain with essential oxygen and nutrients. It relieves cerebral ischemia, or brain tissue damage caused by a lack of oxygen and nutrients, accelerates the structural remodeling of the injured brain, and ultimately promotes the recuperation of neurological function [51]. Promoting angiogenesis has emerged as a promising approach to address central nervous system injuries. The regulation of angiogenesis is dependent upon a plethora of vascular growth factors, with the VEGF as one example, but not exclusively, which facilitates the growth and movement of endothelial cells and also improves vascular permeability [52]. The majority of the angiogenic in vivo effects of the VEGF are the result of the actions of the VEGFR-2. In vitro studies have demonstrated that the VEGFR-2 is involved in the regulation of a number of processes, including the permeability of microvessels, the proliferation, migration and survival of endothelial cells [53]. The VEGFR-2 can transduce VEGF signaling through multiple intracellular signaling pathways [54]. The administration of simvastatin has been demonstrated to promote angiogenesis in both the lesion border zone and the hippocampus following TBI, as well as improving functional recovery. This effect is attributed to activate the VEGFR-2/Akt/endothelial nitric oxide synthase (eNOS) pathway [50]. These observations further support our findings regarding the potential role of lncRNAs in regulating angiogenesis and neural repair. The cytokine IL-10 is involved in numerous pathophysiological processes and plays a pivotal role in the pathogenesis of muscle diseases [55]. The upregulation of IL-10 expression has been demonstrated to prevent muscle atrophy in mice following TBI [55]. There is evidence that the expression of pro-inflammatory molecules can be inhibited by specific drugs in glial cells through the upregulation of IκBα, which is mediated by type 1A phosphatidylinositol-3 kinase (PI3K). Furthermore, it resulted in a reduction in neuronal apoptosis and vascular and blood–brain barrier disruption in the mice brains after TBI, and it demonstrated improvements in memory and motor activity [56]. This finding also supports the potential role of lncRNAs in immune regulation and neuroprotection in our study.

Several limitations of our study should be acknowledged. First, the sample size of N = 3 per group may increase the likelihood of false positives, particularly those unrelated to TBI. Second, although MeRIP-seq has played an important role in identifying m^5^C modifications in RNA, it has limitations in resolution and sensitivity [57]. This method relies on antibody enrichment, which may introduce biases and lacks single-base resolution, potentially leading to false positives or undetected modifications [58]. Future studies could employ more advanced technologies to further validate and complement these findings. Additionally, there are inherent limitations to conducting GO or similar analyses on lncRNAs. Unlike protein-coding genes, lncRNAs often lack well-defined functional annotations, making it challenging to directly interpret their roles [59]. Traditional GO analysis is mainly designed for protein-coding genes, so lncRNA functions are typically inferred indirectly, often based on their proximity to protein-coding genes, target associations, or roles within regulatory networks [60]. This indirect approach can lead to functional enrichment results that are incomplete or less accurate.

In essence, this study presents the first demonstration of altered lncRNA m^5^C methylation following TBI in rats, revealing a new layer of post-transcriptional regulatory changes associated with TBI pathophysiology. By identifying distinct m^5^C methylation patterns in lncRNAs, our findings lay the groundwork for future studies to investigate the specific mechanisms by which these methylation changes impact TBI progression and recovery. This research not only expands our understanding of the molecular landscape of TBI but also highlights the potential of lncRNA m^5^C methylation as a novel biomarker for TBI diagnosis and prognosis. Furthermore, understanding the role of m^5^C-modified lncRNAs in TBI may guide the development of new therapeutic approaches aimed at modulating methylation patterns for enhanced recovery and neuroprotection. Nevertheless, it is crucial to acknowledge that correlation does not imply causation; additional studies are necessary to further investigate the association between lncRNA m^5^C methylation and TBI.

## Figures and Tables

**Table 1:** Top 20 differently m^5^C methylated lncRNAs after TBI.

Chromosome	Transcript id	Gene Name	Log_2_FC	*p*-Value	Regulation
chr2	ENSRNOT00000087639	AABR07013888.1	10.900	7.63 × 10^−10^	up
chr15	ENSRNOT00000083952	LOC102554608	10.680	2.29 × 10^−9^	up
chr9	ENSRNOT00000080726	AABR07067641.1	10.582	2.71 × 10^−9^	up
chr20	ENSRNOT00000082815	Rn50_20_0046.8	10.350	2.27 × 10^−9^	up
chr1	ENSRNOT00000084795	AABR07003007.1	10.282	2.57 × 10^−9^	up
chr8	ENSRNOT00000077688	AABR07069067.1	10.125	2.52 × 10^−9^	up
chr8	ENSRNOT00000091570	AABR07071198.1	9.986	1.39 × 10^−9^	up
chr15	ENSRNOT00000090163	AABR07016947.1	9.979	2.47 × 10^−9^	up
chr1	ENSRNOT00000089632	AABR07006925.1	9.960	2.40 × 10^−9^	up
chr11	ENSRNOT00000087021	AABR07033564.2	9.904	2.26 × 10^−9^	up
chr5	ENSRNOT00000081820	AC110351.2	−8.811	5.05 × 10^−9^	down
chr1	ENSRNOT00000079890	AABR07003304.2	−8.577	4.26 × 10^−9^	down
chr10	ENSRNOT00000078328	Rn50_10_0862.1	−8.412	1.32 × 10^−8^	down
chr4	ENSRNOT00000081779	AABR07060341.2	−8.350	4.65 × 10^−9^	down
chr16	ENSRNOT00000067642	AABR07025328.1	−8.170	7.22 × 10^−9^	down
chr3	ENSRNOT00000085951	AABR07054583.1	−8.126	4.48 × 10^−9^	down
chr14	ENSRNOT00000082693	AC108312.2	−8.098	5.31 × 10^−9^	down
chr6	ENSRNOT00000092708	Rn60_6_0139.1	−8.050	4.68 × 10^−9^	down
chrX	ENSRNOT00000076115	Rn50_X_0744.3	−8.045	1.42 × 10^−8^	down
chr6	ENSRNOT00000092406	Rn60_6_0139.2	−8.024	4.73 × 10^−9^	down

**Table 2:** Top 20 differently expressed lncRNAs after TBI.

Chromosome	Transcript id	Gene Name	Log_2_FC	*p*-Value	Regulation
chr19	ENSRNOT00000087266	AC123500.1	7.239	3.72 × 10^−5^	up
chr20	ENSRNOT00000090814	Rn50_20_0066.11	6.300	4.99 × 10^−3^	up
chrX	ENSRNOT00000072290	AABR07036920.1	6.196	6.73 × 10^−3^	up
chr10	ENSRNOT00000005433	AABR07029847.1	6.093	8.88 × 10^−3^	up
chr6	ENSRNOT00000042226	AABR07064809.1	5.917	1.32 × 10^−2^	up
chr15	ENSRNOT00000079896	AC141168.1	5.721	1.93 × 10^−2^	up
chr4	ENSRNOT00000087525	AC115202.1	5.718	1.94 × 10^−2^	up
chr16	ENSRNOT00000086688	AABR07024814.2	5.657	2.17 × 10^−2^	up
chr1	ENSRNOT00000078400	AABR07005921.1	5.579	2.50 × 10^−2^	up
chr2	ENSRNOT00000090546	AABR07007717.3	5.570	2.53 × 10^−2^	up
chrX	ENSRNOT00000042767	Rn50_X_0232.1	−8.370	3.17 × 10^−9^	down
chr5	ENSRNOT00000092776	Rn60_5_1374.5	−7.050	1.31 × 10^−4^	down
chr19	ENSRNOT00000077876	AABR07043772.1	−6.483	2.77 × 10^−3^	down
chr1	ENSRNOT00000089974	AABR07001623.1	−6.224	5.66 × 10^−3^	down
chr7	ENSRNOT00000086704	AABR07057146.1	−6.123	7.57 × 10^−3^	down
chr11	ENSRNOT00000090855	AABR07034455.1	−6.029	9.31 × 10^−3^	down
chr19	ENSRNOT00000082702	AABR07043106.1	−5.714	1.81 × 10^−2^	down
chr2	ENSRNOT00000089103	AC098750.3	−5.604	2.23 × 10^−2^	down
chr20	ENSRNOT00000089589	Rn60_20_0017.1	−5.573	2.34 × 10^−2^	down
chr2	ENSRNOT00000085759	AABR07008424.1	−5.533	2.64 × 10^−2^	down

**Table 3:** Hyper-methylated and up-regulated lncRNAs.

Chromosome	Transcript id	Gene Name	m^5^C-Log_2_FC	Exp-Log2FC	Regulation
chr10	ENSRNOT00000005433	AABR07029847.1	1.707	6.093	up-up
chr14	ENSRNOT00000050454	Rn50_14_0474.1	9.080	4.309	up-up
chr1	ENSRNOT00000078400	AABR07005921.1	2.042	5.579	up-up
chr15	ENSRNOT00000079896	AC141168.1	2.708	5.721	up-up
chr8	ENSRNOT00000080817	AABR07070926.1	2.310	2.561	up-up
chr17	ENSRNOT00000081442	AABR07026984.1	9.531	3.908	up-up
chr19	ENSRNOT00000087266	AC123500.1	1.470	7.239	up-up
chr13	ENSRNOT00000092264	Lamc2	1.777	4.100	up-up

**Table 4:** Hyper-methylated and down-regulated lncRNAs.

Chromosome	Transcript id	Gene Name	m^5^C-Log_2_FC	Exp-Log2FC	Regulation
chr15	ENSRNOT00000073331	AC109837.1	6.131	−4.240	up-down
chr7	ENSRNOT00000077500	AABR07055333.1	2.051	−1.992	up-down
chrX	ENSRNOT00000080500	AABR07040629.1	1.155	−1.461	up-down
chr3	ENSRNOT00000084353	LOC102551100	3.654	−1.842	up-down
chr1	ENSRNOT00000089974	AABR07001623.1	6.148	−6.224	up-down
chr7	ENSRNOT00000090134	AABR07056330.1	2.320	−1.159	up-down
chr20	ENSRNOT00000092329	Rn60_20_0054.2	2.255	−1.947	up-down

**Table 5:** Hypo-methylated and up-regulated lncRNAs.

Chromosome	Transcript id	Gene Name	m^5^C-Log_2_FC	Exp-Log2FC	Regulation
chr11	ENSRNOT00000075105	Rn50_11_0375.8	−2.507	1.942	down-up
chr8	ENSRNOT00000080817	AABR07070926.1	−2.268	2.561	down-up
chr5	ENSRNOT00000081820	AC110351.2	−8.811	1.238	down-up
chr10	ENSRNOT00000092746	Rn60_10_0621.3	−1.127	3.563	down-up

**Table 6:** Hypo-methylated and down-regulated lncRNAs.

Chromosome	Transcript id	Gene Name	m^5^C-Log_2_FC	Exp-Log2FC	Regulation
chr11	ENSRNOT00000082278	AABR07072264.4	2.236	−2.803	down-down
chr10	ENSRNOT00000086815	AABR07030498.1	−3.437	−1.186	down-down
chr2	ENSRNOT00000088094	AABR07007878.1	−4.022	−1.002	down-down
chr20	ENSRNOT00000092329	Rn60_20_0054.2	−2.647	−1.947	down-down
chr20	ENSRNOT00000092458	Vps52	−1.303	−2.085	down-down

## Data Availability

The sequencing data are uploaded to the NCBI GEO database and are accessible through the GEO Series accession number GSE276398. All relevant samples from this study have been assigned individual GEO sample accession numbers. For more details, please refer to the data link: https://www.ncbi.nlm.nih.gov/geo/query/acc.cgi?acc=GSE276398 (accessed on 8 November 2024).

## Supplementary Materials

The following supporting information can be downloaded at: https://www.mdpi.com/article/10.3390/cimb46120871/s1.
